# Droplets generated from toilets during urination as a possible vehicle of carbapenem-resistant *Klebsiella pneumoniae*

**DOI:** 10.1186/s13756-021-01023-5

**Published:** 2021-10-20

**Authors:** Fabio Arena, Anna Rita Daniela Coda, Valentina Meschini, Roberto Verzicco, Arcangelo Liso

**Affiliations:** 1 Department of Clinical and Experimental Medicine, University of Foggia, Via Luigi Pinto, 71122 Foggia, Italy; 2 IRCCS Don Carlo Gnocchi Foundation ONLUS, Florence, Italy; 3 Department of Medical and Surgical Sciences, University of Foggia, Foggia, Italy; 4 Department of Industrial Engineering, University of Rome ‘Tor Vergata’, Rome, Italy; 5 Maths Division, Gran Sasso Science Institute, L’Aquila, Italy

**Keywords:** Multidrug-resistant bacteria, Toilet, Foam, Prevention, KPC-type carbapenemase

## Abstract

**Background:**

In the health care setting, infection control actions are fundamental for containing the dissemination of multidrug-resistant bacteria (MDR). Carbapenemase-producing Enterobacterales (CPE), especially *Klebsiella pneumoniae* (CR-KP), can spread among patients, although the dynamics of transmission are not fully known. Since CR-KP is present in wastewater and microorganisms are not completely removed from the toilet bowl by flushing, the risk of transmission in settings where toilets are shared should be addressed. We investigated whether urinating generates droplets that can be a vehicle for bacteria and explored the use of an innovative foam to control and eliminate this phenomenon.

**Methods:**

To study droplet formation during urination, we set up an experiment in which different geometrical configurations of toilets could be reproduced and customized. To demonstrate that droplets can mobilize bacteria from the toilet bowl, a standard ceramic toilet was contaminated with a KPC-producing *Klebsiella pneumoniae* ST101 isolate. Then, we reproduced urination and attached culture dishes to the bottom of the toilet lid for bacterial colony recovery with and without foam.

**Results:**

Rebound droplets invariably formed, irrespective of the geometrical configuration of the toilet. In microbiological experiments, we demonstrated that bacteria are always mobilized from the toilet bowl (mean value: 0.11 ± 0.05 CFU/cm^2^) and showed that a specific foam layer can completely suppress mobilization.

**Conclusions:**

Our study demonstrated that droplets generated from toilets during urination can be a hidden source of CR-KP transmission in settings where toilets are shared among colonized and noncolonized patients.

**Supplementary Information:**

The online version contains supplementary material available at 10.1186/s13756-021-01023-5.

## Introduction

Antimicrobial resistance (AMR) is emerging worldwide in hospital and community settings. The World Health Organization (WHO) has recently declared that AMR is one of the top 10 global public health threats facing humanity [1]. It has been estimated that approximately 25,000 patients die each year in the EU from infections caused by multidrug-resistant bacteria (MDR) [2]. The United Nations Ad Hoc Interagency Coordinating Group on Antimicrobial Resistance has recently stated that if AMR is not curbed, in the worst scenario, 10 million people worldwide will die from MDR infections per year by 2050 [3].

Among the most problematic pathogen/antibiotic resistance mechanism combinations is that of carbapenemase-producing Enterobacterales (CPE), which is associated with a high mortality rate.

*Klebsiella pneumoniae* is an especially serious threat for hospitalized patients, patients admitted to long-term acute care facilities and those in rehabilitation settings due to its profile of multidrug resistance and its ability to cause life-threatening infections (mainly of the respiratory system and bloodstream) [4–6].

The major risk factor for carbapenem-resistant *K. pneumoniae* (CR-KP) infection in the hospital setting is prior gut colonization. It has been estimated that approximately 50% of CR-KP infections result from patients’ own intestinal microbiota [7, 8]. However, the dynamics of transmission underlying the acquisition of intestinal colonization by CR-KP in the hospital, long-term acute care and rehabilitation settings are not fully known.

In the hospital setting, without appropriate infection control actions, microorganisms can spread from patient to patient by contact with environmental surfaces [9] or the hands of health care workers [10]. Environmental surface contamination by CR-KP patients appears to be frequent, particularly among superspreaders [11]. Some studies underscore the role of close contact with a colonized patient as a risk factor for CR-KP acquisition, although an explicit definition of “close contact” is lacking [12].

Hospital wastewater is an increasingly recognized reservoir for resistant Gram-negative organisms. Gram-negative bacteria can survive in plumbing (e.g., sink drains, toilets) [13, 14].

A study conducted between 2016 and 2019 in a haematological ward of a French teaching hospital investigated a persistent outbreak of CPE producing OXA-48 carbapenemase and highlighted the role of contaminated toilets in transmission. The incidence of CPE cases declined after intensive toilet cleaning, subsequent replacement with rimless toilets and improved patient hygiene [15].

The role of sink contamination in transmission is more deeply understood, but studies evaluating the role of toilets in the transmission of MDR organisms are lacking. Park et al. recently demonstrated that exposure to CR-KP-colonized/infected patients was associated with environmental (toilet/hopper) CR-KP contamination [13]. Furthermore, it has been shown that a large amount of bacteria or viruses might remain in toilet bowls after flushing due to adsorption to porcelain, with gradual partial elution occurring after multiple flushes [16–18].

Contaminated toilets, therefore, can represent a relevant hidden reservoir of CR-KP in the health care setting. In fact, it is possible that CR-KP contaminating toilets, after being used by a colonized patient, can splash back onto noncolonized people who share the toilet. This is particularly relevant for some health care facilities (e.g., rehabilitation centres and long-term care facilities), which generally have a lower proportion of single bedrooms than acute care hospitals.

While the physical effect of droplet dispersion during flushing has been investigated [16, 17], the dispersion of droplets during urination has not been studied and remains unaddressed in scientific studies. Hence, it is unknown whether droplet dispersion during urination contributes to the spread of microorganisms.

The aim of this study was to assess how urination can mobilize droplets from a toilet contaminated with CR-KP prior to urination. In fact, the hygienic conditions of shared toilets at work, while travelling or even at home can play an important role in the transmission of infectious diseases [19].

We propose a solution for containing the spreading of infectious diseases by shared toilets that involves the use of an innovative foam to control and eliminate the dispersion of droplets generated by the urine flow hitting the bowl walls.

## Material and methods

### Experimental toilet device and demonstration of droplet dispersion during simulated urination

We developed an experimental device comprising a base made of transparent and flexible material that allowed us to change the height of the toilet rim over the water surface and the sloping of the posterior wall to reproduce different models of toilet bowls (Additional file 1: Fig. 1).

**Fig. 1:**
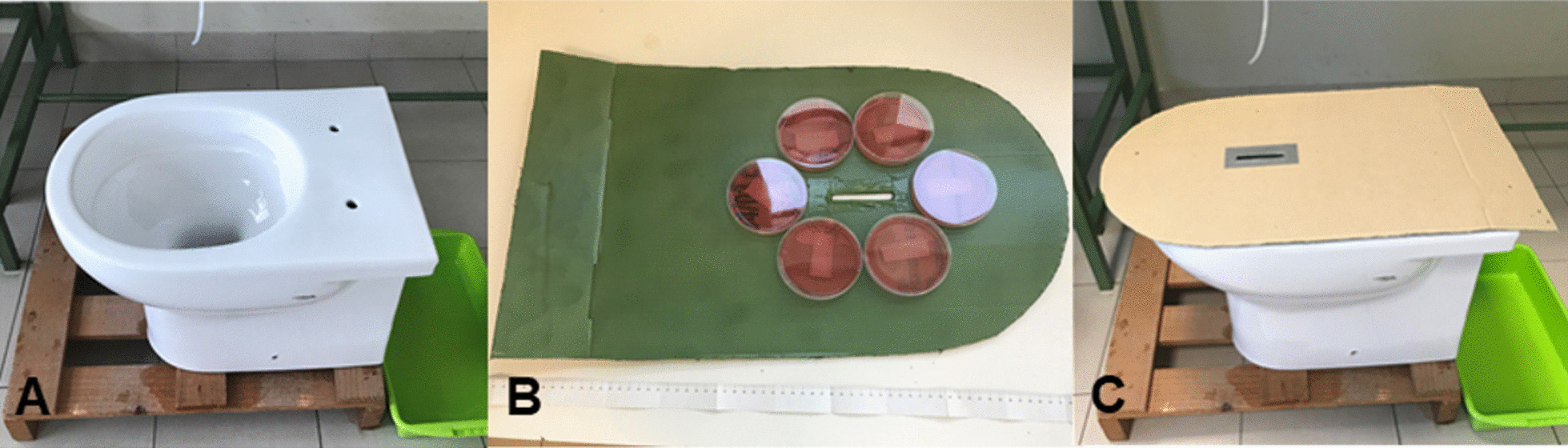
Experimental equipment used to reproduce bacterial contamination: a toilet with 2000 mL of bacteria-containing solution poured into it, a tube (internal diameter: 4 mm) through which to pour 500 mL of sterile saline solution (**A**), a toilet cover fitted six MacConckey agar plates (**B**) affixed to the side facing the inside of the toilet bowl (**C**)

To simulate the formation of drops during urination, we used a device consisting of a plastic tube with a diameter of 4 mm, a tank and a small tap to regulate the water flow. The hydrodynamics of human urination were simulated using a liquid jet at 37 °C (to ensure the same surface tension as urine) with a 20 mL/s flux [20]. The water was coloured with methylene blue in such a way that the droplets produced a visible splatter on white absorbent paper (70 × 70 cm) placed on the toilet seat.

Using high-resolution imaging (36 Mpixel) of the paper surface, acquired by a Nikon D800 camera supported by a tripod, we could analyse and estimate the exact volume and the spatial arrangements of the droplets dispersed by the primary jet and their rebound into the toilet bowl during urination. The software used to postprocess the images was ImageJ (www.imagej.nih.gov/ij), and droplet counting was performed using the Analyse Particles function; this function, however, required a minimum size (minimum to 2 × 2 pixels) for a droplet to be valid and a minimum circularity (ratio of short-to-long axes length: 0.8) to identify a drop as a single droplet rather the coalescence of two or more of droplets.

The characteristic values of colour shades, saturation and brightness were standardized and used for all experiments.

Furthermore, by evaluating the colour intensity of the individual spots in a preliminary calibration process, it was possible to trace the overall volume of dispersed fluid. The various samples were analysed by means of an automated process. The experiments were conducted in the absence and presence of foam in triplicate in 20 different toilet configurations.

### The foam

The foam was a specifically designed chemical formulation containing the following components: a betaine; an anionic surfactant; a dialkyl carbonate; a fatty alcohol with linear or branched alkyl chain, saturated or unsaturated, containing 4 carbon atoms; an ester of a fatty acid with a linear or branched alkyl chain, saturated or unsaturated, containing 4 carbon atoms; a pH regulator; a deodorizing substance and/or a propellant gas. It was also designed to have other features, such as 1) cleaning action, 2) biodegradability for environmental sustainability, 3) stability regardless of the temperature, humidity and ventilation conditions, 4) constant coverage and visibility of the deposited surface, 5) easy elimination with the standard quantity of water used for toilet flushing, and 6) a pleasant appearance and smell. All of its components are commercially available, widely used and known to be nontoxic.

The foam was delivered using a practical sprayer that allowed it to be deposited into the bowl by manually orienting the nozzle. It was sprayed into the toilet bowl until the walls of the bowl and water at the bottom of the toilet was covered by a thick layer (2–3 cm approx.); the foam is characterized by specific persistence and adhesion properties (patent application number: n. 102020000006820).

### Bacterial contamination model

The experiments simulated what happens inside a toilet during urination. We reproduced this effect in the laboratory setting using a standard ceramic toilet bowl (Additional file 2: Fig. 2) with a tube (internal diameter: 4 mm) to simulate urination (Fig. 1A) and a toilet seat cover (made of cardboard) with six culture dishes attached to its underside for the recovery of bacterial colonies (Fig. 1B, C).

**Fig. 2:**
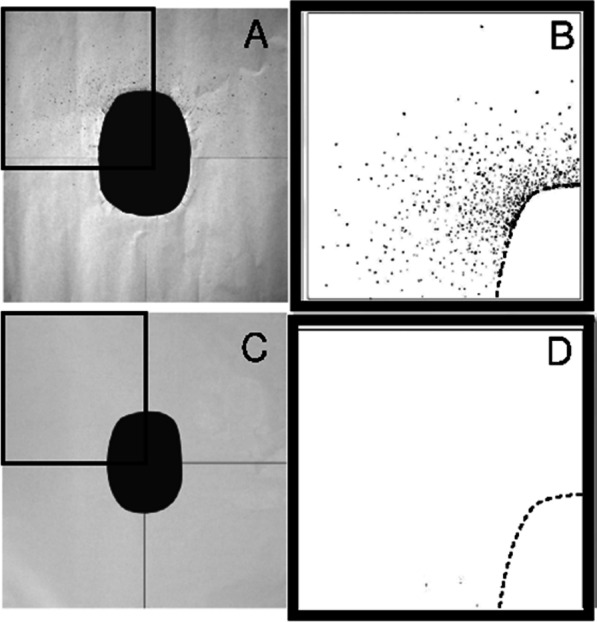
Representative experiment performed without the foam. The droplets that formed during urination are visible on the paper covering the toilet seat (area 70 × 70 cm), and the surface was divided into four quadrants measuring 35 × 35 cm each (**A**). The droplets were counted, and coverage was calculated (n. 1386); the percentage of the surface covered by all of the droplets was 1.71% (**B**). Representative experiment with the foam formulation shows that the foam was able to prevent the mobilization of droplets, and therefore, no drops are present on the paper (**C**); computation shows that 0% of the surface covered with drops (**D**).

Before and after each experiment, the toilet surfaces were carefully manually flushed with sterile water and then cleaned with 70% ethanol.

We simulated the urination process using a beaker containing 500 mL of distilled water at 37 °C and positioned at a height of 2 m and a tube (diameter of 4 mm) with a small tap to open and close the flow. The tube was placed through a small slit in the toilet cover to reproduce a person sitting while urinating. The tube was positioned to orient the liquid flow against the anterior toilet wall for 15 s and against the water surface for another 15 s, with a flow rate of 20 mL/s.

The experiments were performed under three different conditions (A, B and C), each of which was repeated in triplicate:

A) Two litres of sterile saline (negative control, not contaminated with CR-KP) was poured into the toilet, and the liquid jet was produced with 500 mL sterile saline for 30 s.

B) Two litres of bacterial suspension containing 1 × 10^5^ CFU/mL of the bacterial isolate in sterile saline was poured gently into the bowl to uniformly contaminate the internal surfaces of the toilet and fill the bottom. After 30 s, a liquid jet was produced with 500 mL sterile saline for 30 s.

C) Two litres of bacterial suspension containing 1 × 10^5^ CFU/mL of the bacterial isolate in saline was poured gently into the bowl to uniformly contaminate the internal surfaces of the toilet and to fill the bottom; a uniform layer (2–3 cm approx. thickness) of foam was then sprayed onto the internal walls of the toilet, and then a liquid jet was produced with 500 mL sterile saline for 30 s.

The MacConkey agar plates used in each experiment were then incubated in ambient air at 35 ± 2 °C overnight. The microbial load is expressed as recovered CFU/cm^2^. The recovered colonies were identified at the species level by MALDI-TOF mass spectrometry.

### Bacterial isolate

An MDR *Klebsiella pneumoniae* isolate (KP04_2019_UNIFG), obtained in 2019 from blood cultures of a patient with sepsis, was used for the experiments [21, 22].

The KP04_2019_UNIFG Whole Genome Shotgun sequencing project has been deposited at DDBJ/ENA/GenBank under the accession JAFHKY000000000. The version described in this paper is version JAFHKY010000000 [23].

Kleborate software was used for the determination of multilocus sequence typing (MLST) and the content of acquired genes [24].

After overnight growth on MacConckey agar medium, KP04_2019_UNIFG forms round, mucoid, nonhypermucoviscous colonies (negative string test) [25]. The isolate is phenotypically resistant to amikacin, gentamicin, fosfomycin, meropenem, ceftazidime, piperacillin-tazobactam, levofloxacin and colistin and belongs to a widely disseminated high-risk clone (ST101). The KP04_2019_UNIFG genome encodes the *bla*_KPC-3_ and *armA* resistance genes.

### Preparation of the bacterial suspension for toilet contamination

The isolate was grown in LB agar plates aerobically at 35 ± 2 °C overnight. Bacterial colonies were then suspended in saline to reach 0.5 MF, corresponding to approximately 1 × 10^8^ CFU/mL. The suspension was then diluted in 2000 mL of saline to a final bacterial load of 1 × 10^5^ CFU/mL, the bacterial load found in toilet bowl water and greywater [26–28]; similar or even higher inocula were previously used by Baker et al. in a *Serratia marcescens* contamination model [29].

### Evaluation of the antibacterial activity of the foam

After overnight growth on MacConkey agar medium, four or five isolated colonies of KP04_2019_UNIFG were suspended in 2 mL of sterile saline, standardized to a turbidity of 0.5 McFarland standards and inoculated on the dried surface of a Mueller–Hinton agar plate.

To verify the presence of an inhibition zone in the centre of the plate, a small quantity of the foam (approx. 1 cm diameter) was placed on the centre of the plate. The plate was then incubated at 35 °C ± 2 °C in air for 18–24 h and inspected.

## Results

### Different geometrical configurations of toilets cannot prevent droplet formation

Using the previously described experimental toilet bowl with variations in the inclination angle of the solid surfaces (between 40° and 80°, increasing in steps of 10°) and the height of the liquid level inside the bowl (distances between the water-free surface inside the bowl and toilet seat ranging from 30 to 45 cm, increasing in 5-cm steps), we demonstrated the constant production of droplets by the toilet. A typical result obtained during one of the experiments is shown in Fig. 2 and demonstrates traces of secondary droplets on the blotting paper, shown by the number of blue spots occupying a certain surface. In experiments without foam, the number of recovered droplets was 1350 ± 137 (mean value ± standard deviation).

The experiments demonstrated that there were some inclination angles and water levels that better reduced the amount of dispersed droplets, which was strongly dependent on the parameters of the incoming liquid jet (initial height, inclination, flow rate and duration). However, even if some toilet bowl configurations were better than others at reducing droplet generation, they cannot completely eliminate urine dispersion.

### Droplet formation is suppressed by the use of a specific foam

The observation that the generation of droplets is caused by the splashing of the liquid jet against the bowl walls or the water inside the bowl suggested that a possible solution could be provided by modifying the interface conditions at the impact point of the jet. We confirmed that the deposition of a foam layer before urination was an efficient strategy for solving the problem, as it was able to completely suppress the generation of droplets. Upon disappearing, the foam did not leave any visible or unpleasant residue due to its smell and appearance.

### Droplets can be a vehicle for multidrug-resistant bacteria

**Fig. 3:**
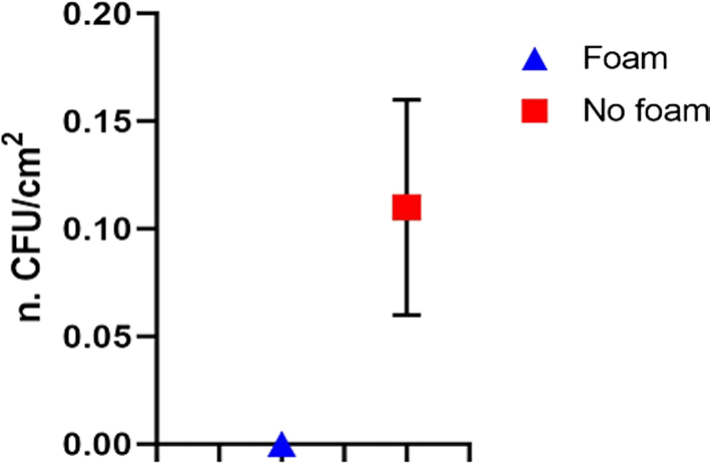
Mean values and standard deviation (CFU/cm^2^) of the colonies collected in the three replicates. The blue triangle represents the experimental conditions in the presence of the foam, and the red square shows the results without the use of the foam

**Fig. 4:**
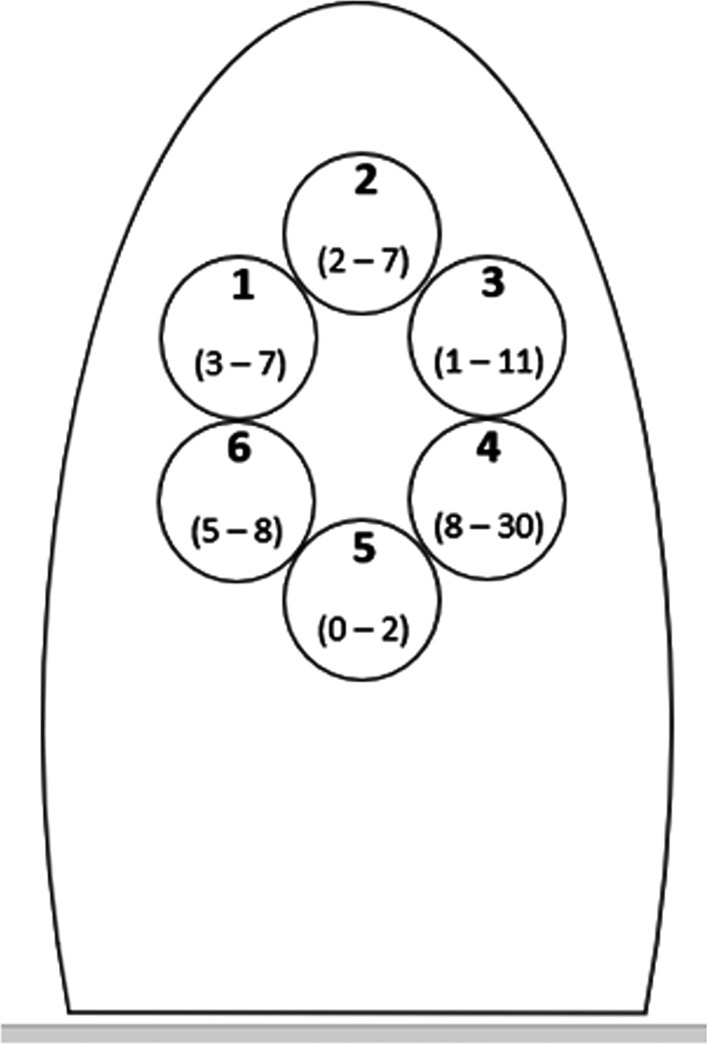
Schematic reproduction of the toilet seat with the six Petri dishes numbered from 1 to 6 (in bold). For each plate, a numerical range is reported in brackets, indicating the number of bacterial colonies recovered, expressed in CFU, for the three replications and the condition in which no foam was used

After overnight incubation, bacterial growth was found in all experiments in Condition B (bacterial contamination without foam). The recovered bacterial colonies were all identified as *Klebsiella pneumoniae*. The mean number of bacterial colonies recovered (Fig. 3) was 0.11 ± 0.05 CFU/cm^2^ (mean value ± standard deviation of the collected colonies, normalized for the total area of the six plates in the three experimental replicates). There was no specific pattern of distribution of the recovered colonies, and the range of recovered colonies for each plate was 0–30 CFU/plate (Fig. 4).

### Foam can trap multidrug-resistant bacteria and has no antibacterial activity

Unlike Condition B, after overnight incubation with the presence of CR-KP suspension, we observed a complete absence of bacterial growth in both the negative control (A, experiments using only sterile saline without CR-KP suspension) and in the condition in which the foam layer was used (C, experiment with CR-KP suspension and the addition of foam).

Regarding the evaluation of the foam’s antibacterial activity, after overnight incubation, no bacterial growth inhibition was observed in the culture medium.

## Discussion

It has been long known that the hygienic conditions of shared bathrooms and toilet seats can play an important role in the transmission of infections [19].

Because microorganisms are not completely removed from toilets by flushing [14–16], in settings where toilets are shared, the risk of infectious agent transmission related to the formation of droplets during urination cannot be ignored.

In fact, the genitourinary mucosa and the perineal cutaneous surface, especially those of people urinating while sitting, can be reached by droplet rebound, which can represent a vehicle for pathogens contaminating the toilet.

In the present work, we demonstrated that i) droplets are generated during urination in virtually all toilet configurations; ii) generated droplets can be a vehicle for CR-KP; and iii) an innovative foam formulation sprayed before each use of the toilet is effective for preventing the generation of droplet rebound during urination.

All these findings can also be considered valid for defecation, which can generate even more abundant splashes.

Using a bacterial strain belonging to a well-known *K. pneumoniae* high-risk clone, we provided a proof of concept that droplets generated during urination can be considered a possible hidden vehicle of CR-KP dissemination in settings (especially health care settings) in which toilets are shared by intestinally colonized and noncolonized patients. This could be of high relevance for settings where patients with intestinal colonization by CR-KP are autonomous and share a room with noncolonized patients (e.g., rehabilitation centres, long-term care facilities, etc.).

For our experiments, we decided to reproduce the microbiological characteristics of greywater and to use a comparable order of magnitude of bacterial contamination to demonstrate the effectiveness of the foam formulation.

Furthermore, according to the literature, droplets produced during urination can also float in the air and reach all surrounding surfaces, including taps, handles, and sinks [16]. Interestingly, droplet generation, and therefore contamination in general, can be completely suppressed by means of foam. The demonstration that bacteria could not escape the foam trap strongly suggests, on physical and fluid dynamics grounds, that smaller particles (viruses, spores) will also be controlled by the foam. However, more experiments, along with properly designed clinical studies, are warranted to demonstrate that microorganisms other than multiresistant bacteria are trapped by the foam.

The use of foams as tools for preventing the spread of MDR bacteria by shared toilets can also be proposed outside the health care setting. Foams offer several important advantages as they can be deposited onto virtually any surface and can be easily flushed away once urination is completed.

They are extremely effective and act on the water at the bottom of the toilet bowl, preventing the formation of splashes due to the direct impact of the water jet not only with toilet walls but also with the water on the bottom of the bowl. Foams are inexpensive and convenient; additionally, their consistency and persistence can be varied within a wide range of values, scents can be easily added to them, they can be made of biodegradable components, and finally, they can be delivered via small, compact spray bottles and carried to almost any location.

An important finding is that the proposed foam is not apparently endowed with an antibacterial effect and therefore should have limited or no effect in terms of selection of resistance and alteration of the bacterial ecology. Our foam was designed to be effective when a low amount of the product was sprayed to form a 2–3 cm thick barrier. The approximate cost of producing a spray can be very reasonable, and therefore, we estimate that a single use would cost a few cents. This compares very favourably with the costs of cleaning practices that ensure disinfection after each toilet use. The spray is very easy to use, and we predict that the use of foam would be widely accepted.

Although the results obtained leave no doubt regarding the effectiveness of the foam, our study has several limitations. In particular, we chose to adopt conditions that simulated a high level of contamination. Further studies with scaled bacterial loads, different bacterial species and comparisons between foam and other devices for limiting the risk of infection (e.g., bleach tablets) will be needed to support our findings.

Overall, there is a global need to improve the sanitation standards of shared toilets and to implement hygiene awareness and education programmes that could reduce the impact of infectious diseases, especially now, in the pandemic era [30]. Current guidelines regarding sanitation in public places have been recently updated [31], and they contain recommendations to frequently implement an enhanced cleaning and disinfection regimen. In this context, the use of foams is more practicable than cleaning a toilet with bleach or other detergents after each use. In fact, since cleaning procedures are costly and labour intensive, it is important to introduce other add-on tools that help reduce the risks of infection when it is not possible to perform cleaning procedures after each use of a toilet.

The use of this foam could be recommended in situations marked by the heavy use of public toilets, including health care, workplaces, transport, culture, and entertainment contexts. Situations of heavy use of shared toilets are most likely while travelling, at large manufacturing or industrial production sites, and at the crowded public events that will soon take place again in the so-called new normal world.

It is hoped that if the results presented here are confirmed in clinical settings, the specifically designed foams could become a novel tool for inhibiting the volatilization of microorganisms, which will have important implications for assuring higher hygienic standards and better preventing the spread of infectious agents in multiple settings.

### Limitations

This was not a clinical study, and we have described laboratory findings only.

## Supplementary Information

**Additional file 1: Figure 1**. Reproduction of a toilet made of a transparent and flexible material (A) with a plastic tube, through which water flows, and absorbent paper to capture droplets (B).

**Additional file 2: Figure 2**. Schematic images representing the standard ceramic toilet bowl used to perform the experiments with bacterial suspensions (measurements are reported in millimetres).

## Data Availability

The datasets used and analysed in the current study are available from the corresponding author on reasonable request.
