# Supplementary figures and images for: Droplets generated from toilets during urination as a possible vehicle of carbapenem-resistant Klebsiella pneumoniae
Source: Antimicrob Resist Infect Control. 2021 Oct 20;10:149. doi: 10.1186/s13756-021-01023-5 (PMC8527778; doi:10.1186/s13756-021-01023-5)

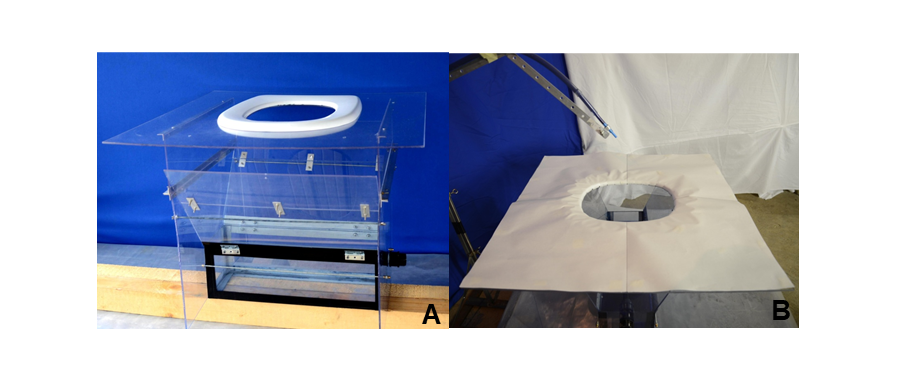

Supplement: Supplementary file 1 — Additional file 1: Figure 1. Reproduction of a toilet made of a transparent and flexible material (A) with a plastic tube, through which water flows, and absorbent paper to capture droplets (B). [file 13756_2021_1023_MOESM1_ESM.tif]

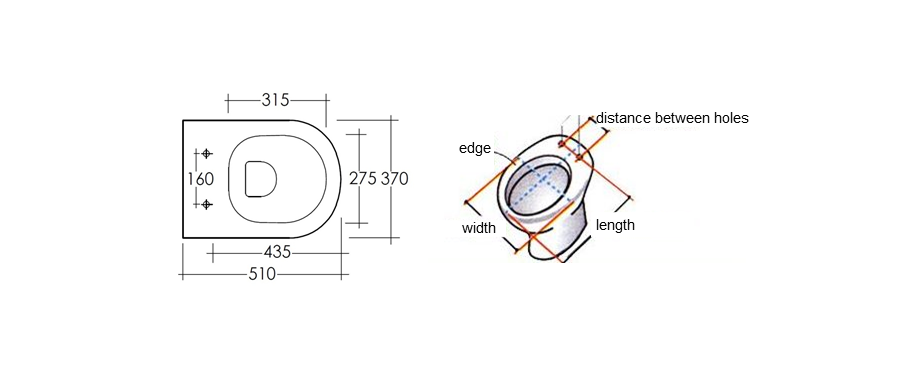

Supplement: Supplementary file 2 — Additional file 2: Figure 2. Schematic images representing the standard ceramic toilet bowl used to perform the experiments with bacterial suspensions (measurements are reported in millimetres). [file 13756_2021_1023_MOESM2_ESM.tif]
